# Biological soil crusts decrease infiltration but increase erosion resistance in a human-disturbed tropical dry forest

**DOI:** 10.3389/fmicb.2023.1136322

**Published:** 2023-04-20

**Authors:** Michelle Szyja, Vincent J. M. N. L. Felde, Sara Lückel, Marcelo Tabarelli, Inara R. Leal, Burkhard Büdel, Rainer Wirth

**Affiliations:** ^1^Molecular Botany (Plant Ecology Group), University of Kaiserslautern, Kaiserslautern, Germany; ^2^Institute of Soil Science, Leibniz University Hannover, Hanover, Germany; ^3^Department of Soil Science, Faculty of Organic Agricultural Sciences, Kassel University, Witzenhausen, Germany; ^4^Departamento de Botânica, Universidade Federal de Pernambuco, Recife, Pernambuco, Brazil

**Keywords:** biological soil crust, tropical dry forest, water infiltration and sorptivity, aggregate stability, regeneration, ecosystem services, ecosystem engineer

## Abstract

Under continuous human disturbance, regeneration is the basis for biodiversity persistence and ecosystem service provision. In tropical dry forests, edaphic ecosystem engineering by biological soil crusts (biocrusts) could impact regeneration by influencing erosion control and soil water and nutrient fluxes, which impact landscape hydrology, geomorphology, and ecosystem functioning. This study investigated the effect of cyanobacteria-dominated biocrusts on water infiltration and aggregate stability in a human-modified landscape of the Caatinga dry forest (NE Brazil), a system characterized by high levels of forest degradation and increasing aridity. By trapping dust and swelling of cyanobacterial filaments, biocrusts can seal soil surfaces and slow down infiltration, which potentially induces erosion. To quantify hydraulic properties and erosion control, we used minidisc-infiltrometry, raindrop-simulation, and wet sieving at two sites with contrasting disturbance levels: an active cashew plantation and an abandoned field experiencing forest regeneration, both characterized by sandy soils. Under disturbance, biocrusts had a stronger negative impact on infiltration (reduction by 42% vs. 37% during regeneration), although biocrusts under regenerating conditions had the lowest absolute sorptivity (0.042 ± 0.02 cm s^−1/2^) and unsaturated hydraulic conductivity (0.0015 ± 0.0008 cm s^−1^), with a doubled water repellency. Biocrusts provided high soil aggregate stability although stability increased considerably with progression of biocrust succession (raindrop simulation disturbed: 0.19 ± 0.22 J vs. regenerating: 0.54 ± 0.22 J). The formation of stable aggregates by early successional biocrusts on sandy soils suggests protection of dry forest soils even on the worst land use/soil degradation scenario with a high soil erosion risk. Our results confirm that biocrusts covering bare interspaces between vascular plants in human-modified landscapes play an important role in surface water availability and erosion control. Biocrusts have the potential to reduce land degradation, but their associated ecosystem services like erosion protection, can be impaired by disturbance. Considering an average biocrust coverage of 8.1% of the Caatinga landscapes, further research should aim to quantify the contribution of biocrusts to forest recovery to fully understand the role they play in the functioning of this poorly explored ecosystem.

## 1. Introduction

Tropical forests are disappearing worldwide mainly due to human-related causes, with seasonally dry tropical forests (hereafter dry forests) particularly suffering from intensified land-use (Miles et al., 2006; Corona-Núñez et al., 2021). Dry forests cover 42% of all tropical habitats, are home to at least one billion people, and maintain the livelihood of millions of people by delivering a wide range of ecosystem services, such as nutrient cycling, primary productivity, food supply, and income generation (Sunderland et al., 2015; Silva and Barbosa, 2017). This dependency, combined with century-lasting human disturbances and intensifying climate change, has made dry forests one of the most impacted ecosystems on the planet (Riggio et al., 2020). To meet the sustainable development goals of the United Nations in these often economically poor environments (Schmerbeck and Fiener, 2015; UN General Assembly, 2015), safeguarding biodiversity and ensuring the continuous provision of ecosystem services are necessary. Both rely on the forest’s ability to regenerate from human disturbance (i.e., forest resilience; Chazdon, 2008).

The ability of dry forests to regenerate is strongly driven by edaphic legacies derived from disturbance, notably a reduced water availability and an increased soil erosion risk (Becknell and Powers, 2014). By influencing vegetation and soil development (Poorter et al., 2019) both edaphic properties are tightly linked to biodiversity, ecosystem functioning, productivity, and ecosystem service provision (Quijas et al., 2019). Water availability is a key limiting factor in dry forests and directly influences seed production, seed bank composition, germination, seedling survival, and plant growth (Vieira and Scariot, 2006). However, the strong dependency of the local population on forest goods and services has intensified water limitations. Conversion of 50% of dry forests worldwide has drastically altered the infiltration capacity of their soils, as agricultural practices compact and seal soils with long-lasting negative effects on water availability (Leite et al., 2018). The subsequent drought stress has aggravated seedling mortality and selected towards a set of resprouting species, leading to biotic homogenization in dry forests (Barros et al., 2021; Vanderlei et al., 2022). The limited infiltrability of soils under disturbance not only reduces the local water availability, including ground-water recharge and soil water holding capacity (Abdallah et al., 2021). It also increases the chances of overland flow run-off, flooding, and erosion (Ludwig et al., 2005; Bradshaw et al., 2007). Soil erosion, which refers to the removal of unstable soil surfaces mostly by wind and water, can cause drastic and potentially irreversible changes for ecological and socioecological systems on the local, regional, and continental scale (Flores et al., 2020). As soil erosion reduces the water holding capacity and therefore water availability of the soil it can directly influence all water-related limitations (Andraski and Lowery, 1992). Soil erosion is also accompanied by dust production and deposition in streams (Belnap et al., 2011), crop damage (Neff et al., 2005), and degraded soil quality (Karlen and Rice, 2015). Based on rainfall duration, magnitude, and intensity, dry forests are facing a moderate risk to rainfall-induced erosion (Panagos et al., 2017). However, the highly erodible soils of many dry forests (Rito et al., 2017), a high degree of leaf deciduousness during the dry season, and a reduced leaf litter cover caused by livestock grazing (Pfister et al., 1988) leave dry forest soils unprotected against the physical impact of raindrops during high-intensity rains, inducing a strong land degradation process (Bruijnzeel, 1990). Furthermore, soil erosion can be directly induced by human land use, such as slash-and-burn agriculture and shifting cultivation, two of the major disturbance agents in dry forests (Blackie et al., 2014; Borrelli et al., 2017). Moreover, rainfall and subsequent soil erosion over deforested areas can remove the seedbank required for forest regeneration (García-Fayos et al., 2010). Even under sufficient seed-supply soil erosion can obstruct forest recovery by creating a negative, self-reinforcing feedback loop, due to a lack in nutrient supply and capture (Flores et al., 2020). Aggravatingly, soil formation rates are very often so low that once the nutrient-rich dry forest topsoil layer has been removed by erosion, opportunities for forest restoration or regeneration are highly limited (Lal, 1990). The disturbance-derived reduced water availability in combination with increased soil erosion represent effective environmental filters selecting for floristic subsets of stress-tolerant plant species and thus modify successional trajectories during regeneration (Flores et al., 2020). Continuing disturbances over decades can lead to severe soil degradation (Celentano et al., 2017), forcing the local population to expand their activities deeper into old-growth forests. This further destabilizes and degrades dry forest soils, also with consequences for soil water availability and plant species composition. As a result, large parts of dry forests are being turned into steppes or deserts (Vieira et al., 2015). Despite the functional links between soil health and forest regeneration, studies on soil and water-related ecosystem services have been largely neglected in dry forests, even though they could be the decisive factor for their resilience (Calvo-Rodriguez et al., 2017; Quijas et al., 2019).

After disturbance, soils are often bare and degraded and are covered by primary colonizers during secondary succession like biological soil crusts (biocrusts) (Pointing and Belnap, 2012; Szyja et al., 2019). Biocrusts are a complex community of organisms, composed of cyanobacteria, green microalgae, bryophytes, lichens, fungi, heterotrophic bacteria, and archaea, as well as representatives of several invertebrate animal groups, living in or on the uppermost millimeters of the soil (Belnap et al., 2016). Poikilohydry and other adaptations have enabled them to survive harsh abiotic conditions like high solar irradiance, extreme temperatures, and water limitations (Bowker, 2007), making them key organisms during primary and secondary succession in drylands, including dry forests (Szyja et al., 2019). Biocrusts are regarded as ecosystem engineers, as many of their soil-related effects influence the establishment, survival, and productivity of vascular vegetation (Havrilla et al., 2019). Specifically, their influence on soil water availability and erosion control could affect dry forest regeneration. More precisely, biocrusts influence soil hydrology *via* changes in soil porosity, aggregation, organic matter content, and water repellency, thereby affecting soil water infiltration, hydraulic conductivity, and retention (Chamizo et al., 2012; Rodríguez-Caballero et al., 2013). Additionally, by changing soil surface roughness and water storage capacity, they affect the water residence time on the surface and water velocity during runoff (Rodríguez-Caballero et al., 2012, 2015). Erosion-reducing effects result from the fact that biocrusts entrap soil particles *via* organic exudates (extracellular polysaccharide sheaths; EPS) and filamentous structures. Thereby they stabilize the soil surface (Rossi et al., 2018), promote soil aggregate formation and increase erosion resistance against wind and water (Belnap et al., 2014; Chamizo et al., 2015). The biocrust effect on edaphic properties increases with biocrust developmental stage, which can be related to an increase in biocrust biomass, thickness, and EPS production, which stabilize the soil (Rossi et al., 2018). Another stabilizing factor is the biocrust-contributed soil organic carbon (SOC), which increases with biocrust succession and can be composed of increasingly more water repellent substances (Drahorad and Felix-Henningsen, 2013). As anthropogenic disturbance in biocrusts leads to a retrogressive shift to an earlier successional stage human impact can impair their ecosystem service provision (Kuske et al., 2012). It is plausible to assume that the edaphic ecosystem services provided by biocrusts could shape dry forest resilience. Their impact on soil water infiltration and stability has the potential to influence two of the most important edaphic properties necessary for successful dry forest regeneration, water availability and erosion control. Given that ecosystem engineering impacts vary with environmental context, with a greater effect in more arid or resource poor environments (Wright et al., 2006), biocrusts could be highly significant for semi-arid, often resource poor dry forests (Rodríguez-Caballero et al., 2018), but also highly threatened by the ever-increasing human disturbance in these forests. Despite such potentially far-reaching consequences for forest resilience, the ecosystem engineering effect of biocrusts in dry forests is yet to be investigated, both in their natural state and during regeneration after disturbance.

The most continuous dry forest of the New World, the Caatinga in northeast Brazil, has been transformed by intense human impact since the 16th century and currently suffers from slash-and-burn agriculture and free-ranging livestock farming (Silva and Barbosa, 2017). The remaining forest stands, even if denoted as natural reserves and protected sites, often experience chronic anthropogenic disturbance like firewood, fodder, and timber collection (Arnan et al., 2018). The continuous opening of the Caatinga old-growth dry forest has transformed it into a dynamic mosaic of active and abandoned fields and forests of different successional stages (Tabarelli et al., 2017). Under such conditions, biocrust occurrence is facilitated, e.g., on abandoned fields, with an average cover of 8.1%, and locally over 50% (Szyja et al., 2019). Considering that large parts of the Caatinga are composed of structurally unstable Arenosols with a low water holding capacity, biocrusts may provide essential ecosystem services related to erosion control and soil water availability, especially during times of low vegetation cover, i.e., the dry season. However, the vulnerability of biocrusts to small- and large-scale disturbances together with soils of low aggregate stability might result in an inability of the biocrust to fulfil their pedological and ecological roles (Chamizo et al., 2015). In this context, the Caatinga offers an excellent opportunity to investigate the ecological role played by biocrusts in dry forests.

The aim of this study was to evaluate the impact of biocrusts on soil water infiltration and erosion control in the Caatinga dry forest and discuss its implications for ecosystem resilience and vegetation regeneration. For this, we measured sorptivity, unsaturated hydraulic conductivity, and water repellency as infiltration parameters and soil surface penetration resistance and wet aggregate stability as erosion related parameters. To assess the effect of human disturbance on these biocrust-mediated ecosystem services, the investigations were conducted at two sites with contrasting disturbance levels: an active cashew plantation (grazed by cattle) and an abandoned pastureland with regenerating Caatinga vegetation. We hypothesize that biocrust presence reduces infiltrability compared to biocrust-free soil, with a stronger water-repellent effect in the later successional biocrust due to a higher amount of soil organic carbon. We also expect that biocrusts will increase soil penetration resistance and wet aggregate stability, even under disturbed conditions, as there they can be the main soil stabilizers due to a reduced vegetation cover, but that disturbance will lessen this stabilizing effect due to successional setbacks, which confer weaker erosion resistance (Belnap and Büdel, 2016).

## 2. Materials and methods

### 2.1. Study area

**Figure 1:**
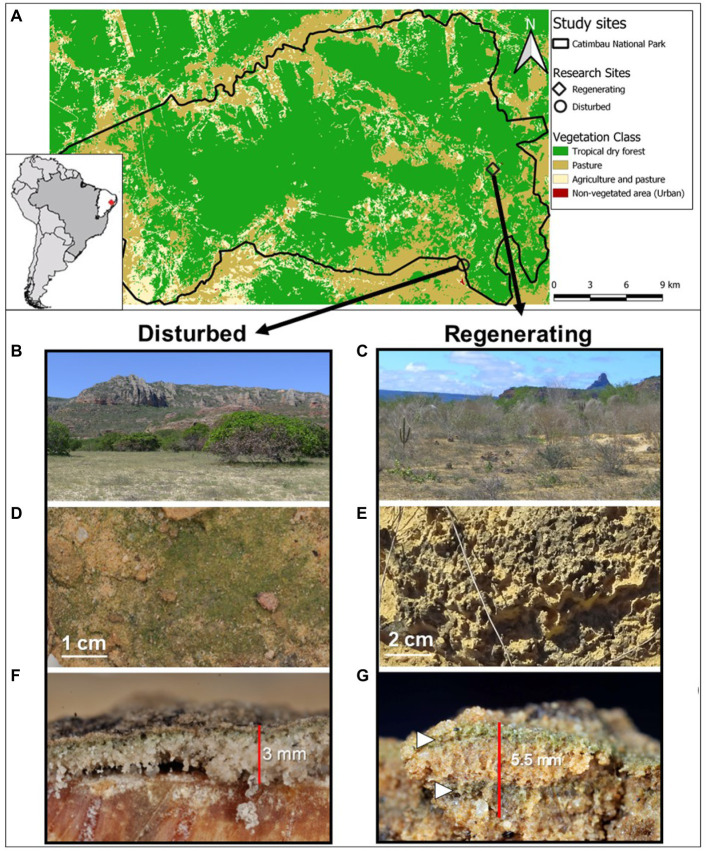
Case study sites within the Catimbau National Park, northeastern Brazil **(A)** including their associated biocrust communities. Overview of the two study sites at the very end of the dry season, an actively disturbed cashew plantation **(B)** and an abandoned, regenerating farm **(C)**. Closeups of the cyanobacteria-dominated biocrusts with a smooth **(D)** and a pinnacled surface **(E)**. Vertical cut through both biocrusts depicting a thin biocrust layer in the disturbed site **(F)** and a thick, possibly double-layered biocrust (white arrows) on the regenerating site **(G)**.

This study was carried out in the Catimbau National Park (8°24′00″ and 8°36′35″ S; 37°00′30″ and 37°10′40″ W; Figure 1) in the Caatinga dry forest, northeast Brazil. The predominant soil type is sedimentary, deep, infertile, and acidic (pH 4.5) Arenosols, with occasional Planosol and Vertisol presence (Rito et al., 2017). The climate is semi-arid (precipitation to potential evapotranspiration ratio < 0.65) with an annual temperature of 23°C (Sampaio, 1995). Precipitation ranges from 480 to 1,100 mm y^−1^ within the National Park and is concentrated between March to July, with a pronounced dry season (≤50 mm month^−1^) from August to February (Sociedade Nordestina de Ecologia (SNE), 2002) and high spatial and temporal variations, including droughts lasting over a year (Rito et al., 2017). Despite being declared a National Park in 2002, the 607 km^2^ encompassing area still contains scattered villages with about 1,000 inhabitants and consists of a vegetation mosaic of different physiognomies, ranging from crop fields and pastures to low-statured old-growth dry forests, dominated by Euphorbiaceae and Fabaceae (Rito et al., 2017). Open areas dominated by Cactaceae and Bromeliaceae, arbustive Caatinga, and second-growth forests of varying ages are also an important, persistent, and expanding component of the Catimbau National Park (Tabarelli et al., 2017). All these vegetation types are suffering from chronic anthropogenic disturbance, e.g., firewood and forage collection, and livestock browsing (Arnan et al., 2018).

All experiments were carried out at two case study sites along the disturbance gradient of the Catimbau National Park, representing contrasting biocrust successional stages (for a more detailed description of the study sites and organismal composition, see Szyja et al., 2019; Table 1; Figure 1). The early successional site (“disturbed site”) was an actively managed cashew plantation and pasture site with smooth, light cyanobacterial biocrusts. The late successional site (“regenerating site”) was a former pastureland, on which a young secondary forest dominated by shrub vegetation developed following abandonment *ca.* 40 years ago. This site occasionally suffered from trampling, with dark, pinnacled cyanobacterial biocrusts, including scattered bryophytes and lichens. According to their particle size distribution using the USDA textural triangle, the disturbed site was classified as sandy soil and the regenerating site as loamy sand. Measurements were done on the most representative biocrusts at both sites: light cyanobacteria in the disturbed site and dark cyanobacteria in the regenerating site, with bryophyte- and lichen-dominated biocrusts deliberately excluded from the analysis. All *in situ* investigations were carried out in March before the rainy season of 2017 (soil penetration resistance), and 2018 (water infiltration). The samples for the aggregate stability measurements were collected in March and May of 2017. All measurements and samples were taken after 1 week without rainfall, to ensure dry soil conditions, as unsaturated hydraulic conductivity and soil penetration resistance are both dependent on soil water content or water tension (Vaz et al., 2001; Chamizo et al., 2015; Supplementary Table S1).

**Table 1:** Description of the two case study sites, including area, mean annual precipitation, soil texture, disturbance history, biocrust coverage, successional stage, and roughness, bulk density, porosity, and soil organic carbon (SOC) in biocrusts and bare control soil.

	Disturbed		Regenerating	
Area [ha]	3.96		2.64	
Mean annual rainfall [mm]	736		645	
Soil texture	Sandy		Loamy sand	
Disturbance history	Active plantation and pasture for cattle and chicken; annual weeding		Abandoned farmland 40 years ago	
Biocrust coverage [%]	7.6		45.11	
Biocrust successional stage and roughness	Early, light cyanobacteria-dominated, smooth		Late, dark cyanobacteria-dominated; occasional bryophytes and lichens, pinnacled	
	**Biocrust (*n* = 45)**	**Bare soil (*n* = 16)**	**Biocrust (*n* = 45)**	**Bare soil (*n* = 16)**
Bulk density [g cm^−3^]	1.28 (±0.12)^A^	1.54 (±0.04)^B^	1.16 (±0.08)^C^	1.45 (±0.08)^B^
Porosity [%]	52 (±1.9)^A^	42 (±3.2)^B^	56 (±2.1)^C^	45 (±4.1)^B^
SOC in first cm [g kg^−1^]	10.67 (±4.5)^A^	6.04 (±2.3)^B^	19.82 (±4.0)^C^	8.7 (±4.5)^AB^

Standard deviations are in parentheses and statistically significant differences are denotated in letters. All variables stem from Szyja et al. (2019).

### 2.2. Field methods

Measurements in the field were carried out on biocrust patches (≥40 cm^2^) with no apparent recent disturbance. Additionally, control soils devoid vegetation were sampled that were impacted by the local disturbance pressure (topsoil, 0–5 cm).

#### 2.2.1. Soil hydrology

Soil hydrology was investigated using minidisc-infiltrometry following Lichner et al. (2007) and Keck et al. (2016). At both sites, *n* = 25 biocrust and *n* = 20 control patches were investigated, with a minimum distance of 5 m between patches. Per patch, the measured parameters were unsaturated hydraulic conductivity, sorptivity, and repellency index, obtained by simultaneously employing two minidisc-infiltrometers (Decagon Devices, Pullman, United States) filled with either water or ethanol, at 20 cm distance to each other. Measurements were carried out under a pressure head value of *h_0_* = −4 cm to exclude macro pores from the infiltration process and prevent rapid infiltration into the coarse sandy soil. Prior to each measurement, a thin layer of medium textured, non-repellent local sand from the regenerating site (sieved to <1 mm particle size) was applied on the biocrust surface to ensure full contact of the infiltrometers’ steel disk (Decagon Devices, 2012). Each infiltration measurement was video-recorded for 2 min using a small compact camera (Panasonic Lumix DMC-LX5). During video analysis in the laboratory infiltration intervals of 10 s were chosen for further analysis. All measurements were carried out under dry conditions. To calculate the water content of the sample at the beginning of the experiment, soil samples up to 4 cm deep were collected on five biocrust and five respective control soils and dried at 105°C in a drying oven until no change in weight could be detected (after approximately 72 h; Supplementary Table S1). Calculations followed Zhang (1997) which work well for dry soil (for calculations details and formulas see Supplemental material).

#### 2.2.2. Soil penetration resistance

Soil penetration resistance and vertical stratigraphy of biocrusts (up to 4 cm deep) was examined using an electronic micro penetrometer (EMP; Drahorad and Felix-Henningsen, 2012). The probe tip and shaft geometries were according to Felde et al. (2018), using a 3 mm diameter plain sided, flat tipped 90° probe, with 39 μm step sizes per measurement, which enables maximum resolution of the soil stability profile. Measurements were taken on soils covered by biocrusts in *n* = 10 (disturbed) and *n* = 8 (regenerating) patches. Each patch had a corresponding control patch within a maximal distance of 1 m, and with a lateral distance of 50–100 m between biocrust-control pairs. Due to the moisture-dependency of soil penetration resistance (Chamizo et al., 2015), the measurements were done under dry and wet conditions, where wet equals measurement immediately after the application and infiltration of 10 ml water cm^−2^, applied with a spray bottle. This amount of water is sufficient to reach the optimal water content for cyanobacterial dominated biocrusts (Szyja et al., 2018). To measure the water content for each replicate (Supplementary Table S1), soil samples up to 4 cm deep (equals the depth of the EMP probe) were collected once before and after spraying with water for biocrusts and controls. The samples were dried in a drying oven at 80°C until no change in weight was detected anymore (after 72 h). Higher temperatures could not be achieved due to field site limitations in 2017, which likely resulted in slight underestimations of the water content. For wet biocrusts and control soils, a moisture correction factor was added to the calculation of penetration resistance (see Supplemental material). With it the penetration resistance was adjusted to a common volumetric water content of 0.10 m^3^ m^−3^. Soil texture affects penetration resistance measures considerably (Vaz et al., 2001), therefore the correction was applied separately for each site. Each biocrust replicate and control plot contained six measurement spots, the first three measurements under dry, the last three under wet conditions, with a lateral distance of at least 3 cm to avoid interference between the adjacent observations (Drahorad and Felix-Henningsen, 2012). This totaled in 108 penetration resistance curves for biocrusts and control plots each, of which half were on wet and half on dry soil. The near-surface peak values of the penetration resistance curves were read out as the mean maximum penetration resistance of the biocrust and was compared to the penetration resistance value at the corresponding depth of the control soil. As the peak disappeared in wet biocrusts, the same depth as in the dry penetration resistance curves was analyzed for the wet samples.

### 2.3. Laboratory methods

The aggregate stability measurements were carried out *ex situ* on *n* = 6 biocrust samples per site, with each sample taken at a distance of at least 10 m. If present, aggregates of the control soil did not survive transportation and therefore, no control samples were investigated in this analysis. All samples were tested for aggregate stability using wet sieving (Yoder, 1936) and raindrop simulations (McCalla, 1944).

#### 2.3.1. Wet sieving

For wet sieving 5 g (±0.5 g) of air-dried biocrust samples of uniform initial size (between 8 and 16 mm) were put onto a sieving tower consisting of six sieves with mesh sizes of 8 mm, 4 mm, 2 mm, 1 mm, 0.5 mm, and 0.25 mm. The biocrusts were put on the 8 mm sieve and slowly wetted to saturation for 30 min by only just touching the water surface. After saturation, the sieving tower was raised and lowered under water at a lifting height of 4 cm and at a frequency of 0.5 Hz for 10 min. After being dried for 14 h at 105°C, the mass percentage of each size fraction was calculated. Aggregate stability was expressed as the geometric mean weight diameter, which describes the average log-normalized biocrust aggregate size after the mechanical and hydrological stress from the sieving process. The higher the geometric mean weight diameter, the more stable the aggregate (Kemper and Rosenau, 1986; for calculation details and formulas see Supplemental material). Parallel to the wet sieving, the residual water content of each sample was determined to then be corrected for in the calculation of the water stable aggregates.

#### 2.3.2. Raindrop simulation

To investigate the effect of raindrop impact on the disintegration of biocrust aggregates, a raindrop simulation was carried out. Individual biocrust aggregates were broken down to a defined size of 4–5 mm (Chamizo et al., 2018). Next, specimen were chosen at random and individually placed on a sieve with 3.14 mm mesh size underneath a burette filled with distilled water. The generated drops fall directly and at a constant rate (2 Hz) onto the aggregate until it has been rinsed through the sieve. For each biocrust, 20 repetitions were measured due to high variability of individual values (Koepf, 1956). The fall height of the droplets was 1 m, and their diameter 4 mm. To determine the mass of a droplet, 50 drops were collected in a beaker, weighed, and divided by the drop sum to calculate the average weight of a drop. For better comparability with other studies (even with different drop sizes and drop heights), the required number of drops is converted into the cumulative kinetic energy necessary to destroy the biocrust aggregates (Zhao et al., 2014; for calculations see Supplemental material).

### 2.4. Data analysis

All statistical analyses, except for the general linear mixed model (GLMM), were done in Statistica (Statistica, version 10, StatSoft, Inc., Palo Alto, CA, United States). Prior to all analyses, the data were tested for normal gaussian distribution (Shapiro–Wilk test), and for homogeneity of variance (Levene test). The data for hydrological measurements were square-root transformed to fit normality. To test whether biocrust presence and human disturbance were influencing soil infiltration three two-factorial ANOVAs were applied, where (1) unsaturated hydraulic conductivity, (2) sorptivity, and (3) repellency index were analyzed for biocrust effect (biocrust vs. bare soil), disturbance level (disturbed vs. regenerating), and their interaction term (biocrust*disturbance). To ensure comparability of datasets, the water contents of (4) the infiltration measurements and (5) the dry soil penetration resistance measurements were analyzed for the same effects using two-factorial ANOVAs. All significant analyses were followed by a Tukey *post-hoc* test. To investigate whether the biocrust repellency index correlates with the SOC content of the samples, a Pearson’s correlation test was performed, independently for biocrust and control soils. The repellency index values for both sites were pooled into one value, so that only one biocrust and one control soil correlation was performed. The SOC data for both sites was taken from Szyja et al. (2019). To test if biocrusts had a higher penetration resistance value than the surrounding control soil, each location (disturbed and regenerating) was independently statistically investigated. The data were square transformed prior to analysis to fit normal gaussian distribution. For each location a GLMM was performed, where mean maximum penetration resistance was analyzed for biocrust effect (biocrust vs. bare soil), water content (dry vs. application of 10 ml of water per cm^2^), and its interaction term (biocrust*water). Plot was used as the random factor. The GLMM was run in the R 4.1.1. programming language environment, using the packages nlme, lme4 and MuMIn. To test whether the geometric mean weight diameter of the regenerating and disturbed site differed, a one-way ANOVA was performed. To further test if the aggregate stability differed between biocrusts at both sites (regenerating and disturbed) the kinetic energy released upon raindrop impact necessary to break apart biocrust aggregates was analyzed using the non-parametric Mann–Whitney U-test, due to the data not following a normal gaussian distribution.

## 3. Results

**Figure 2:**
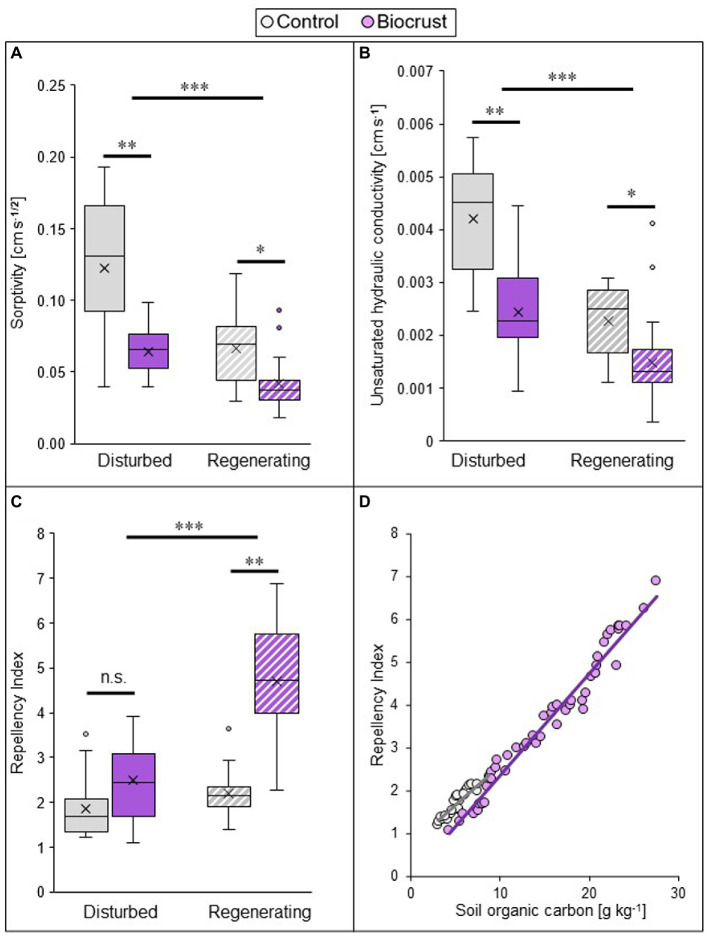
Biocrust and disturbance effects on soil hydrological parameters of two study sites in the Caatinga dry forest, NE Brazil. Sorptivity **(A)**, unsaturated hydraulic conductivity **(B)**, and repellency index **(C)** values measured at a pressure head of *h_0_* = −4 cm, and the relationship between repellency index and soil organic carbon content **(D)** of biocrusts (purple) and bare soil patches (control, grey) in a disturbed (filled in boxes) and regenerating (striped boxes) site in the Caatinga, NE Brazil. For **(A–C)** boxes represent the interquartile distance between the first and third quartile, the horizontal line the median, the cross the statistical mean, and the antennas the next closest point from an outlier within the data set. *n* = 25 for biocrusts and *n* = 20 for controls. Black bars and asterisks describe statistical differences within groups (control vs. biocrust) per site and between groups (disturbed vs. regenerating); *p* < 0.05 = *; *p* < 0.01 = **, *p* < 0.001 = ***. *Post hoc*-test results can be found in Table S2. For **(D)**, data from both case study sites were pooled (*n* = 50 for biocrusts and *n* = 25 for control soils). SOC data taken from Szyja et al. (2019). Pearson’s *r* = 0.98; *p* < 0.001 for biocrust and *r* = 0.92; *p* < 0.0001 for control.

Biocrust presence substantially reduced infiltration, by lowering sorptivity and unsaturated hydraulic conductivity, but increasing water repellency of the soil (Figures 2A–C; Supplementary Table S2, statistical results in Supplementary Table S3). In combination, these values point towards a strong reduction of water input into deeper soil layers by biocrust coverage of the soil surface. This negative biocrust effect on infiltration was consistent between sites, but the disturbance regime exerted a strong impact on all hydrological parameters, with differences already between control soils but also between biocrusts. Comparing the two sites (i.e., disturbance pressure), the lowest absolute values for sorptivity were found in the regenerating site, where they were 54 and 85% lower compared to the disturbed site in both biocrusts and biocrust-free control soils, respectively. The same was true for unsaturated hydraulic conductivity, with values 64 and 84% lower in biocrusts and biocrust-free control soils, respectively (Supplementary Tables S2, S3). This indicates that the biocrust-induced decrease of water uptake into deeper soil layers is larger under regenerating conditions. Within sites, the actual ecosystem impact of the biocrust was largest in the disturbed sites. This is reflected by a reduction of sorptivity by 48% in the disturbed site and by 37% in the regenerating site, compared to the control. Correspondingly, unsaturated hydraulic conductivity decreased by 42 and 35%, respectively. The opposite was found for the repellency index, which was highest in the regenerating site and got significantly increased by biocrust presence only in this site (increase of 115%). Under disturbed conditions biocrusts did not influence the repellency index, which was similar in the early successional biocrust and the biocrust-free control soil (Supplementary Table S2). Soil repellency was strongly and positively related with SOC, for both biocrusts and control soils (Figure 2D).

**Figure 3:**
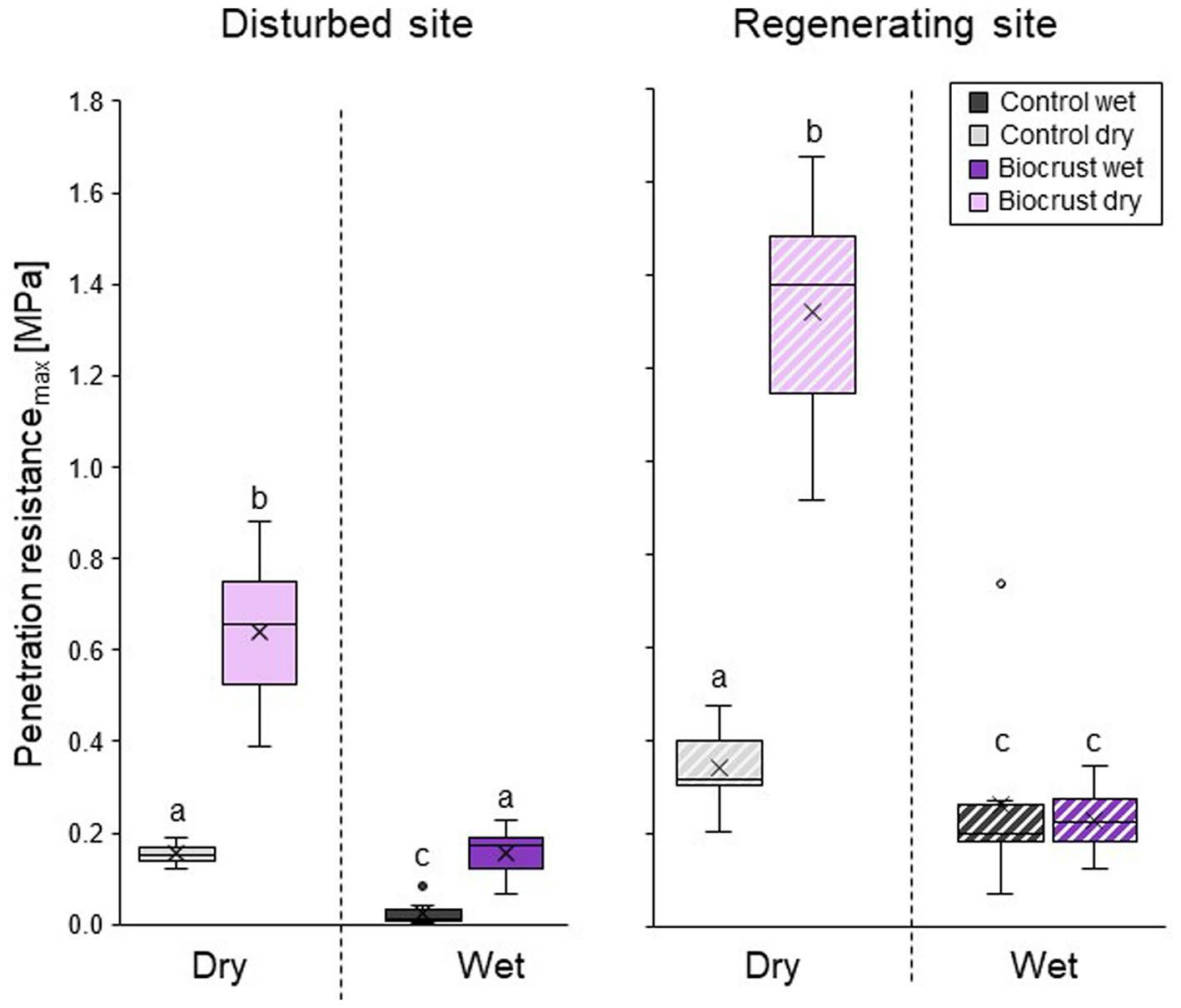
Mean maximum penetration resistance of biocrusts (purple) and bare soil patches (control, grey), in disturbed (filled in boxes) and regenerating (striped boxes) Caatinga dry forest, NE Brazil. Penetration resistance was measured under field dry and wet (water content 0.10 m^3^ m^−3^) soil conditions. Boxes represent the interquartile distance between the first and third quartile, the horizontal line the median, the cross the statistical mean, and the antennas the next closest point from an outlier within the data. *n* = 10 for disturbed and *n* = 8 for regenerating site for biocrusts and control each. Lowercase letters indicate statistical differences between all measurement conditions per site.

**Figure 4:**
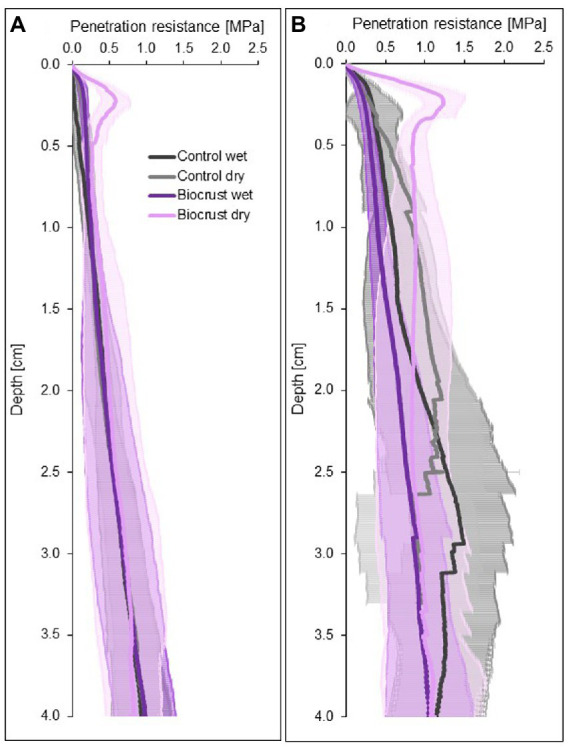
Four-centimeter-deep penetration resistance curves of biocrusts (purple) and bare soil patches (control, grey) at disturbed **(A)** and regenerating **(B)** sites in the Caatinga dry forest, NE Brazil. Penetration resistance was measured under field dry and wet (water content 0.10 m^3^ m^-3^) soil conditions. Lines represent mean penetration resistance values (*n* = 10 for disturbed and *n* = 8 for regenerating site for biocrusts and control soils each), while antennas represent standard deviations.

Dry biocrust presence increased maximum penetration resistance, independent of site (Figure 3; Table 2) and only showed one layer of increased penetration resistance at a depth of *ca.* 2–3 mm (Figure 4). This is indicative of only one biocrust layer within the soil profile at both sites. The soil stabilizing effect was 4.1-fold at the disturbed site (control_dry_: 0.16 ± 0.02 MPa; biocrust_dry_: 0.64 ± 0.17 MPa) and 3.9-fold at the regenerating site (control_dry_: 0.34 ± 0.09 MPa; biocrust_dry_: 1.32 ± 0.26 MPa) and thus independent of the disturbance regime. Wetting reduced penetration resistance values for all sites. In the disturbed site the biocrusts remained harder than the biocrust-free soils, while the difference disappeared in the regenerating site. The observed mean maximum penetration resistance values under wet conditions were similar in biocrusts (0.16 ± 0.05 MPa for disturbed and 0.23 ± 0.07 MPa for regenerating), despite differences in biocrust species compositions and successional stages. It is important to note that the softening of the biocrust under wet conditions does not indicate a lower protection against water-induced erosion, which was tested for with the aggregate stability and raindrop simulation measurements. At the disturbed sites, vertical penetration resistance profiles were more homogeneous showing less variation on penetration resistance at any depth and for any soil surface compared to the regenerating site (Figure 4). The maximum penetration resistance of biocrusts peaked at similar soil depths in both sites (0.22 ± 0.04 cm for disturbed; 0.24 ± 0.05 cm for regenerating).

**Table 2:** Generalized linear mixed models for mean maximum penetration resistance dependency in MPa on biocrusts of different successional stage, disturbance regimes, and water content.

Response variable	Effect	DF	*F*	*p*	*R*^2^m	*R*^2^c
Mean maximum penetration resistance [MPa] disturbed site (*n* = 30)	Biocrust	1; 27	599.14	**<0.001**	0.89	0.94
	Water content	1; 27	28.29	**<0.001**		
	Biocrust*Water	1; 27	609.73	**<0.001**		
	Random Factor	9; 27	9.69	**<0.001**		
Mean maximum penetration resistance [MPa] regenerating site (*n* = 24)	Biocrust	1; 21	180.68	**<0.001**	0.72	0.85
	Water content	1; 21	194.61	**<0.001**		
	Biocrust*Water	1; 21	346.14	**<0.001**		
	Random Factor	9; 21	13.03	**<0.001**		

Investigated were early successional biocrusts of a disturbed site, and late successional biocrusts on a regenerating site. Mean maximum penetration resistance was analyzed for biocrust effect (biocrust vs. bare soil), water content (dry vs. water content of 0.10 m^³^ m^−3^), and its interaction term (biocrust*water). Plot number was used as the random factor. Significant effects are in bold (*p* ≤ 0.05); DF, degree of freedom; F, effect value; *p*, value of *p*; *R*^2^m, marginal *r* squared; *R*^2^c, conditional *r* squared.

**Figure 5:**
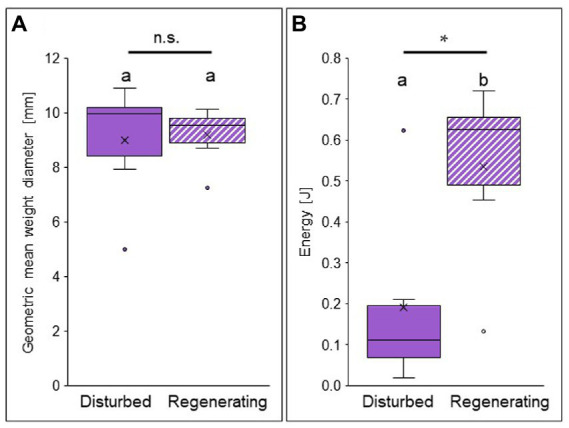
Aggregate stability against water erosion in biocrusts at opposite ends of the disturbance spectrum (disturbed site = filled in bars, and regenerating site = striped bars) in the Caatinga dry forest, NE Brazil. Geometric mean weight diameter of biocrust fragments after wet sieving in mm **(A)** and kinetic energy [Joule] input by raindrops necessary to break apart a biocrust aggregate **(B)**. Boxes represent the interquartile distance between the first and third quartile, the horizontal line the median, the cross the statistical mean, and the antennas the next closest point from an outlier within the data. *p* < 0.05 = *; n.s. = not significant; *n* = 6 each. For statistical results see Supplementary Tables S3, S4.

Aggregate stability, i.e., the protection against water erosion, did not differ between the biocrusts of the disturbed and the regenerating site when analyzing geometric mean weight diameter (Figure 5A). This value describes the stability of biocrust aggregates against mechanic and hydraulic stress and was similar between both sites (9.18 ± 1.05 mm in the regenerating site, 9.00 ± 2.19 mm in the disturbed site; Supplementary Table S3). In contrast, when investigating the effect of raindrop impact, i.e., the vertical kinetic energy transferred onto the biocrust until breakage, a strong difference was observed between the biocrust successional stages (Figure 5B). In the regenerating site, the kinetic energy by raindrop impact necessary to break apart a biocrust aggregate was significantly (almost three times) higher than for the disturbed biocrust (0.54 ± 0.22 J in the regenerating site, 0.19 ± 0.22 J in the disturbed site; Supplementary Table S4).

## 4. Discussion

The low infiltration on biocrusts indicates a reduced water input into the soil under both disturbed and regenerating conditions. The lowest infiltration rates are measured for well-developed dark cyanobacteria-dominated biocrusts found in regenerating vegetation sites, although the biocrust-induced ecosystem impact is largest in more open sites subjected to human disturbance. In constrast, biocrust presence drastically increases protection against water-induced soil erosion not only during regeneration but also under disturbance and for different soil textures. Stability increases considerably with progression of biocrust succession, but early successional biocrusts already form stable aggregates against water erosion on sandy soils even under ongoing disturbance. Despite their low stability against raindrop impact, this result suggests that biocrusts confer protection to dry forest soils even in the worst combination of land-use and soil conditions with a very high soil erosion risk. The findings suggest that biocrusts may take on some of the ecosystem functioning roles otherwise held by vascular plants, influencing dry forest resilience and regeneration.

Despite earlier controversial findings, our results are in accordance with previous meta-analyses (Chamizo et al., 2016; Eldridge et al., 2020). They reinforce the idea that biocrusts limit the infiltration of water to greater depths in sandy soils, and that their infiltration-reducing effect increases with biocrust successional stage (Warren, 2003; Bowker et al., 2008). All three investigated infiltration parameters of the studied Caatinga biocrusts were within the lower range reported for cyanobacteria-dominated biocrusts on sandy soils (Drahorad et al., 2013; Lichner et al., 2018; Guan and Liu, 2021). Water infiltration was consequently strongly reduced by sorptivity and unsaturated hydraulic conductivity, but only moderately by water repellency (with repellency index values in biocrusts in literature ranging from 1.9 to 210; *cf.* Keck et al., 2016). Our results also confirm that biocrusts protect mobile sediments from water-induced soil erosion (Chamizo et al., 2017), with later successional stages delivering higher soil protection (Bowker et al., 2008). Although their protective effect decreases under disturbance the stability conveyed by early successional biocrusts against soil erosion even under disturbed conditions could explain the unexpected result of Leite et al. (2018), who reported no difference in erosion rates between recently abandoned fields and old-growth Caatinga forest. Our stability values were all within ranges previously reported for biocrusts on sandy soils (Zhao et al., 2014; Chamizo et al., 2018; Felde et al., 2018), while slightly towards the lower end under disturbance. Except for geometric mean weight diameter, which had significantly higher values for biocrusts at both sites than previously reported (2.51 ± 0.11 mm, Kakeh et al., 2018), pointing towards an extraordinarily high aggregate stability against water erosion. This remarkably high stability, however, might be due to differing sampling methods employed, as in our study only the biocrust itself (3–5.5 mm) was investigated while Kakeh et al. (2018) investigated the first 5 cm of soil, introducing more soil material to the sample and “diluting” the effect of the biocrust. All penetration resistance measurements were dominated by a single peak of maximum resistance, which showed that the biocrust top layer did not develop on older buried soil surfaces, as was described in Felde et al. (2018). As expected, penetration resistance was reduced after disturbance, which was also described for biocrusts from South Africa (Dojani et al., 2011). The fact that the disturbed site had more uniform penetration resistance is indicative of the loss of soil structure and a higher structural homogeneity after disturbance. The peak of maximum penetration resistance disappeared under wet conditions, which is in accordance to the results of previous studies, that also showed a decrease of penetration resistance under wet conditions (Lapen et al., 2004; Chamizo et al., 2015). However, since this is the first study with high-resolution depth-dependent penetration resistance data of dry and wet biocrusts, it remains unclear if the pattern of completely disappearing penetration resistance peaks of wet biocrusts will replicate in other ecosystems with different edaphic and climatic conditions.

Much of the effects of biocrusts on soil hydrology can be attributed to EPS production. For example, low infiltration rates on biocrusts are likely caused by the blocking of soil matrix pores upon wetting and the subsequent swelling of EPS (Belnap, 2006; Fischer et al., 2010). Biocrust EPS production could also be responsible for the strong water repellency of the late successional biocrusts at the regenerating site. Biocrust hydrophobicity was related to an increasing SOC content (Figure 2D), and up to 75% of SOC is stored as EPS in biocrusts (Mager, 2010). The ability of cyanobacteria-dominated biocrusts to stabilize sandy soils is also connected to EPS production. It functions as a binding matrix between filamentous cyanobacteria and soil particles, leading to soil particle aggregation (Guhra et al., 2019) and dust capture (Danin and Ganor, 1991), which cements the upper layer of the soil (Mazor et al., 1996) and stabilizes the surface (Hu et al., 2002). Specifically, the species *Microcoleus vaginatus* and *Scytonema* sp. have a very high protective effect against wind and water erosion, respectively and only need low biomass to confer protection (Hu et al., 2002). Both were found throughout the Catimbau National Park and were present even under heavy disturbance (Szyja et al., 2019), which explains the erosion protection even of early successional biocrusts under disturbance. The observed increase of biocrust impact on edaphic properties with biocrust succession is related to gains in biocrust biomass, thickness, SOC content and EPS production (Bowker et al., 2008; Rossi et al., 2018). However, biocrust impacts can also be shaped by disturbance, with opposing effects based on biocrust succession and disturbance type (Faist et al., 2017). Acute trampling of the early successional biocrust could increase infiltration compared to untrampled biocrusts, due to the destruction of biocrust integrity and disruption of biocrust-created soil aggregates. In contrast and counterintuitively, trampling of the late-successional cyanobacteria-dominated biocrust could decrease infiltration, due to drastic changes in pore geometry (Felde et al., 2014) and pore clogging by biocrust fragments left in place (Faist et al., 2017). This biocrust succession-based disturbance effect can explain the strong limitation of infiltration of the late successional biocrust compared to the early successional biocrust, although the disturbances at the studied sites were not only acute but also chronic and long-term. Furthermore, disturbance can lead to a loss of fine soil particles to which nutrients are bound, including stabilizing SOC, increasing soil erosion (Hagemann et al., 2017). Additionally, site-specific underlying soil properties (e.g., texture, pore connectivity, bulk density) can significantly shape biocrust effects. They influence biocrust protection against erosion and can change or even override the biocrust effect on infiltration. Firstly, the formation of stabilizing biocrusts is dependent on the soil texture, as coarse textured soils produce thin and less stable biocrusts than fine textured soils (Rozenstein et al., 2014; Chamizo et al., 2015). Secondly, the loamy sand of the regenerating site naturally supports lower infiltration rates than the sandy soil of the disturbed site (Warren, 2003). Thirdly, the higher amount of fine soil particles of the regenerating site is more prone to physical crusting and soil compaction, both reducing infiltration but potentially increasing erosion (Drahorad and Felix-Henningsen, 2013; Chamizo et al., 2015). Consequently, the biocrust and biocrust-free loamy sand of the regenerating site promote a higher erosion protection and lower infiltration. The impact of soil properties is also reflected in the *relative* infiltration parameters per site, where the reduction in hydraulic properties was stronger in the early successional biocrust of the disturbed site, despite a higher infiltration rate. In combination with the lower water holding capacity and rapidly attained wilting point of the sandy substrate (Souza et al., 2020), the disturbed site may induce a fast onset of drought stress for vascular vegetation. The water availability in the disturbed site is therefore probably lower than in the regenerating site, despite higher hydraulic properties. The present study does not allow a quantitative assessment of texture effects, because controlled laboratory experiments that eliminate all other factors would be necessary for this (Rozenstein et al., 2014).

Biocrusts represent a surface runoff source in dry forests due to their reduced soil water infiltration, although the strength of this reduction is likely dependent on seasonality and soil moisture (Chamizo et al., 2012; Keck et al., 2016). Under the assumption that the reduced water infiltration is a stable ecosystem engineering effect, the patchy distribution of the biocrust community creates small-scale source-sink patterns of water- and nutrient availability within the landscape (Cantón et al., 2020). It is plausible to assume that matter fluxes from biocrusted, vegetation-free spots, e.g., abandoned fields, to deposition sites can generate preferential growth conditions and facilitate regeneration as a higher water availability is directly correlated to increased plant recruitment in dry forests (Vieira and Scariot, 2006). Since water redistribution in the Caatinga is naturally rare (de Figueiredo et al., 2016), this would be a particularly impactful ecosystem service. However, the high water infiltration and low water repellency of the sandy Caatinga soils can lead to biocrust-induced runoff being lost before it reaches vegetated patches, creating a resource deficit within the system (Ludwig et al., 2005). Additionally, the smooth surface of early successional biocrusts can potentially increase runoff velocity due to their higher connectivity of runoff pathways, promoting erosion despite its aggregating function (Belnap and Büdel, 2016). The reduced water intake into soils below cyanobacteria-dominated biocrusts can be counterbalanced by their greater soil moisture retention due to blocking of surface pores (Eldridge et al., 2020) and the strongly reduced water vapor diffusion that leads to lower evaporation rates (Benard et al., 2019). These processes support the existence of hotspots of maximum productivity, nutrient content, and microbial activity in dryland soils (Whitford, 2002), which directly benefit from the biocrust-induced restriction of water percolation into greater depths. Additionally, biocrusts can break up self-reinforcing erosion feedback-loops by reducing erosion directly and by increasing and protecting the local nutrient content (Barger et al., 2006; Flores et al., 2020). Erosion control can further help to sustain the life quality of the local population by preventing dust deposition in water sources (Belnap et al., 2011), increasing cropland productivity (Neff et al., 2005), and protecting the seedbank and seedlings (García-Fayos et al., 2010). Currently large parts of dry forests are threatened by desertification caused by unsustainable anthropogenic land exploitation (Miles et al., 2006; Vieira et al., 2015). However, biocrusts have been proven to be useful tools for nature-based dryland restoration and to combat land degradation and desertification (Dadzie et al., 2022; Maggioli et al., 2022). Their influence could become even more important, since dry forests will face dramatic changes and climate extremes, particularly an increase in drought stress for vascular plants (Torres et al., 2017). The following decline in plant biomass (Vieira and Scariot, 2006) would increase the risk for erosion of unprotected soils. Under such circumstances, biocrusts could be the last barrier against land degradation and the decisive factor for dry forest resilience. Edaphic ecosystem engineering by biocrusts must therefore be considered an underexplored key process for ecosystem functionality in anthropogenically disturbed dry forests.

## 5. Conclusion

This study shows that biocrusts reduce water infiltration and at the same time protect against water-induced soil erosion in a human-impacted tropical dry forest. To our knowledge, this ecosystem type was investigated for the first time (Weber et al., 2022). Combined with the high rainfall intensity during the rainy season, the marked decrease in infiltration through biocrusts might be a key factor for surface runoff. Despite showing high runoff parameters, both biocrusts were stable against water-induced erosion, although disturbed biocrusts conferred lower protection against raindrop erosion. The combination of lower erosion protection and reduced water infiltration under disturbance could negatively impact vascular plant establishment and productivity. In contrast, during regeneration biocrusts also decreased water infiltration but protected the most critical dry forest soil layer against soil erosion, which might increase the resilience of this ecosystem. Biocrusts have the potential to reduce land degradation, although their associated ecosystem services can be depleted by disturbance such as grazing. Considering an average biocrust cover of 8.1%, and locally more than 50% (Szyja et al., 2019), biocrusts could be particularly important local ecosystem engineers. Future studies should aim to investigate if biocrust-induced ecosystem service provision is universal across dry forests and independent of the strong seasonality. Related to erosion protection, future studies should focus on how biocrusts can be used as a soil protective agent against erosion for the reversion of the desertification process, and how climate change will impact their ecosystem service provision.

## Data availability statement

The datasets presented in this study can be found in online repositories. The names of the repository/repositories and accession number(s) can be found at: www.figshare.com; DOI to access data directly: https://doi.org/10.6084/m9.figshare.21561183.v1.

## Author contributions

MS, RW, BB, and VF conceived and planned the experiment. MS and SL carried out the experiments with help from RW, BB and VF. MS and SL analyzed the data. BB, RW, IL, MT, and VF contributed to the interpretation of the results. MS took the lead in writing the manuscript. RW, MT, IL, and BB supervised the project. All authors provided critical feedback and helped shape the research, analysis, and manuscript.

## Funding

This study was funded by the Coordenação de Aperfeiçoamento de Pessoal de Nível Superior (CAPES project ID: 88881.030482/2013-01), the Conselho Nacional de Desenvolvimento Científico e Tecnológico (CNPq-PELD project ID: 403770/2012-2) and by the German-Brazilian PROBRAL program (CAPES process 88881.030482/2013-01; DAAD project ID: 57413496) to RW, IL, and MT.

## Conflict of interest

The authors declare that the research was conducted in the absence of any commercial or financial relationships that could be construed as a potential conflict of interest.

## Publisher’s note

All claims expressed in this article are solely those of the authors and do not necessarily represent those of their affiliated organizations, or those of the publisher, the editors and the reviewers. Any product that may be evaluated in this article, or claim that may be made by its manufacturer, is not guaranteed or endorsed by the publisher.
