# Supplementary material for: Biological soil crusts decrease infiltration but increase erosion resistance in a human-disturbed tropical dry forest
Source: Front Microbiol. 2023 Apr 20;14:1136322. doi: 10.3389/fmicb.2023.1136322 (PMC10157249; doi:10.3389/fmicb.2023.1136322)
Supplement: Supplementary file 1 [file Data_Sheet_1.docx]

Supplementary Material

# Water content, hydrological parameters and statistical analyses

Table S1: Water content (WC; m³ m^-^³) of the dry biocrusts and bare control soil at the beginning of the infiltration measurements and during the dry penetration resistance (PR_dry_) measurements. Wet samples were set to a defined water content and not shown here. Standard deviations are in parentheses and statistically significant differences are denotated in letters.

|  | Disturbed | | Regenerating | |
| --- | --- | --- | --- | --- |
|  | **Biocrust** | **Bare Soil** | **Biocrust** | **Bare Soil** |
| WC_Infiltration_ [m^3^ m^-3^] | 0.029 (± 0.0025) ^A^ | 0.030 (± 0.0035) ^A^ | 0.027 (± 0.0029) ^A^ | 0.030 (± 0.0011) ^A^ |
| WC PR_Dry_ [m^3^ m^-3^] | 0.015 (± 0.0026) ^A^ | 0.016 (± 0.0051) ^A^ | 0.012 (± 0.0027) ^A^ | 0.015 (± 0.0026) ^A^ |

Table S2. Biocrust and disturbance effects on soil hydrological parameters of two study sites in the Caatinga dry forest, NE Brazil. Repellency index (RI); sorptivity (S); unsaturated hydraulic conductivity (K); at a pressure head of *h_0_* = –4 cm. With n = 25 for biocrusts and n = 20 on bare soil (control); conducted under field-dry conditions; standard error in parentheses; significant differences (p < 0.05) indicated with letters among columns.

| **Site** | **Treatment** | **RI** | **S (-4)**  **[cm s^-1/2^]** | **K (-4)**  **[****cm s^-1^]** |
| --- | --- | --- | --- | --- |
| **Disturbed** | Biocrust | 2.5 (± 0.17) ^A^ | 0.064 (± 0.003) ^A^ | 0.0024 (± 0.00016) ^A^ |
|  | Control | 1.9 (± 0.14) ^A^ | 0.122 (± 0.011) ^B^ | 0.0042 (± 0.00024) ^B^ |
| **Regenerating** | Biocrust | 4.7 (± 0.23) ^B^ | 0.042 (± 0.004) ^C^ | 0.0015 (± 0.00017) ^C^ |
|  | Control | 2.2 (± 0.12) ^A^ | 0.066 (± 0.005) ^A^ | 0.0023 (± 0.00015) ^A^ |

Table S3: Two-factorial ANOVA results for the effects of disturbance and biocrust presence on water content (1) at the beginning of the infiltration and (2) during the dry penetration resistance measurements, as well as on (3) unsaturated hydraulic conductivity, (4) sorptivity, and (5) repellency index. One-way ANOVA result for biocrust succession effect on geometric mean weight diameter. Investigated were early successional biocrusts of a disturbed site, and late successional biocrusts on a regenerating site. Significant effects are in bold (p ≤ 0.05); DF, degree of freedom; F, effect value; P, p-value.

| **Response Variable** | **Effect** | **DF** | **F** | **P** |
| --- | --- | --- | --- | --- |
| **Water content Infiltration** | Disturbance | 1; 16 | 0.08 | 0.93 |
|  | Biocrust | 1; 16 | 1.52 | 0.24 |
| **Water content dry penetration resistance** | Disturbance | 1; 32 | 0.335 | 0.57 |
|  | Biocrust | 1; 32 | 0.103 | 0.75 |
| **Unsaturated hydraulic conductivity** | Biocrust | 1; 86 | 48.32 | **< 0.001** |
|  | Disturbance | 1; 86 | 61.07 | **< 0.001** |
|  | Biocrust * Disturbance | 1; 86 | 7.03 | **0.01** |
| **Sorptivity** | Biocrust | 1; 86 | 45.85 | **< 0.001** |
|  | Disturbance | 1; 86 | 41.30 | **< 0.001** |
|  | Biocrust * Disturbance | 1; 86 | 7.52 | **0.007** |
| **Repellency Index** | Biocrust | 1; 86 | 75.82 | **< 0.001** |
|  | Disturbance | 1; 86 | 49.25 | **< 0.001** |
|  | Biocrust * Disturbance | 1; 86 | 26.71 | **< 0.001** |
| **Geometric mean weight diameter** | Biocrust | 1; 10 | 0.032 | 0.86 |

Table S4: Results of the Mann-Whitney *U*-test to examine if the kinetic energy by raindrop impact necessary to break biocrusts differs depending on biocrust successional stage. n = disturbed site = regenerating site = 6. Significant effects are in bold (p ≤ 0.05). U, U-value; z, Z score; P, p-value.

| **Response variable** | **Effect** | **U** | **z** | **P** |
| --- | --- | --- | --- | --- |
| **Kinetic energy by raindrop impact** | Biocrust successional stage | 5.00 | -2.00 | **0.045** |

# Formulas to calculate sorptivity, hydraulic conductivity, repellency index

Calculations for unsaturated hydraulic conductivity, sorptivity, and the repellency index following Zhang (1997; **Eq. 1**):

$I=C_{1}(h_{0})t^{1/2}+ C_{2}(h_{0})$ (Eq. 1)

With *I* being the cumulative infiltration and *C_1_ (h_0_)* and *C_2_* *(h_0_)* being functions of pressure head *h_0_*. The unsaturated hydraulic conductivity for the soil *k(h_0_)* at a pressure head *h_0_* ≤ 0 can be calculated as (**Eq. 2**):

$k(h_{0})= \frac{C_{2}(h_{0})}{A_{1}}$ (Eq. 2)

With *A_1_* being constant. The unsaturated hydraulic conductivity *k_r_* (-4 cm), reduced due to soil water repellency, was then estimated from the cumulative infiltration data *I = f(t)* according to equations 1 and 2, using A_1_ = 0.89 for sandy soil and A_1_ = 1.84 for loamy sand and a pressure head *h_0_* = -4 cm from Table 2 in the Minidisk Infiltrometer user’s manual (Decagon Devices, 2012).

At early measurement times of less than 180 s, the sorptivity *S* can be found as the slope of the plot (Clothier et al., 2000; **Eq. 3**):

$I \approx St^{1/2}$ (Eq. 3)

The sorptivity of water *S_w_(h_0_)* and of 96.5% ethanol *S_e_(h_0_)* measured in the field at a pressure head *h_0_* = -4 cm and estimated according to **Eq. 3** can be used to calculate the repellency index. The RI of water was then calculated from (Hallet et al., 2001; **Eq. 4**):

$R=1.95 S_{e}(h_{0})/S_{w}(h_{0})$ (Eq. 4)

# Formula to calculate moisture correction factor

To calculate the soil penetration resistance (PR) of biocrusts, a moisture correction factor must be added (Busscher et al., 2003; **Eq. 5**):

$PR=R_{i}+\left( \partial R_{i}/\partial\theta_{i} \right)\left( \theta_{c}-\theta_{i} \right)$ (Eq. 5)

Where ∂R_i_/∂θ_i_ equals the first derivative of the linear regression between R_i_ (penetration resistance measured in the field) and θ_i_ (soil volumetric water content measured in the field for each sampling site), and θ_c_ as the volumetric water content to which the values are adjusted to.

# Formulas used to calculate geometric mean weight diameter

For wet sieving the residual water content for each sample was determined to then be corrected for in the calculation of the water stable aggregates. For this purpose, 1 g of the biocrust sample was weighed in a crucible and dried at 105 °C for 24 hours. The water content of the biocrust samples was calculated as (**Eq. 6**):

$WC \left[ \% \right]=\frac{WM-DM}{DM*100}$ (Eq. 6)

Where *WM* [g] equals the weight of the wet sample and *DM* [g] equals the dry mass of the sample. Using this equation, the weight of the aggregates can be corrected for the residual water content (**Eq. 7**; Castro Filho et al., 2002):

$WSA_{i} \left[ \% \right]=\left( \frac{DM_{i}-{DM}_{0.25 mm}-sand}{{DM}_{g}-sand} \right)*100$ (Eq. 7)

With *i* being the respective aggregate fraction (16-8, 8-4, 4-2, 2-1, 1-0.5, 0.5-0.25 mm); *DM_i_* [g] being the dry mass of the respective aggregate fraction (*i*) after sieving and *DM_g_* [g] being the dry mass of the corrected total aggregate weight before sieving. Aggregate stability was then expressed using the geometric mean weight diameter (GMD; Van Bavel, 1949; Kemper & Rosenau, 1986) using these equations (**Eq. 8, 9**):

$w_{i} \left[ \% \right]= \frac{{DM}_{i}}{{DM}_{g}}$ (Eq. 8)

$GMD \left[ mm \right]=\exp\left\{ \frac{\sum w_{i}ln\bar{x}_{i}}{\sum w_{i}} \right\}$ (Eq. 9)

With *w_i_* [g] being the ratio of the weight of the respective aggregate fraction (*i*) after the sieving to the corrected total weight of the aggregate sample before sieving and $\bar{x}_{i}$ [%] being the mean diameter of an aggregate fraction (*i*).

# Formulas used to calculate kinetic energy by raindrop impact

The following equations were used to calculate the kinetic energy by raindrop impact necessary to destroy biocrust aggregates (**Eq. 10 and Eq. 11**; Zhao et al., 2014):

$v\left[ ms^{-1} \right]=4.8\left[ d\left( 1-e^{-\frac{0.85h}{d}} \right) \right]^{0.5}$ (Eq. 10)

With *v* [m s^-1^] being the velocity of an average droplet, *d* [mm] being the diameter of the droplet, and *h* [m] equal to the height of fall of the droplet.

$E\left[ J \right]= \frac{1}{2}v^{2}*\sum m_{Droplet}$ (Eq. 11)

With *m_Droplet_* [kg] being the total mass of the droplets necessary for destruction.

# References

Busscher, W. J., & Bauer, P. J. (2003). Soil strength, cotton root growth and lint yield in a southeastern USA coastal loamy sand. Soil and tillage research, 74(2), 151-159.

Castro Filho, C., Lourenço, A., de F. Guimarães, M., & Fonseca, I. C. B. (2002). Aggregate stability under different soil management systems in a red latosol in the state of Parana, Brazil. Soil and Tillage Research, 65(1), 45–51.

Clothier, B. E., Vogeler, I., & Magesan, G. N. (2000). The breakdown of water repellency and solute transport through a hydrophobic soil. Journal of Hydrology, 231, 255-264.

Decagon Devices (2012). Minidisk Infiltrometer User’s Manual. Decagon Devices, Inc., Pullman, USA, 22 pp.

Hallett, P. D., Baumgartl, T., & Young, I. M. (2001). Subcritical water repellency of aggregates from a range of soil management practices. Soil Science Society of America Journal, 65(1), 184-190.

Kemper, W. D., & Rosenau, R. C. (1986). Aggregate Stability and Size Distribution. In A. Klute (Hrsg.), SSSA Book Series (S. 425–442). Soil Science Society of America, American Society of Agronomy.

Van Bavel, C. H. M. (1950). Mean weight-diameter of soil aggregates as a statistical index of aggregation. Proceedings. Soil Science Society of America, 1949, 14, 20-23.

Zhang, R. (1997). Determination of soil sorptivity and hydraulic conductivity from the disk infiltrometer. Soil Science Society of America Journal, 61(4), 1024-1030.

Zhao, Y., Qin, N., Weber, B., & Xu, M. (2014). Response of biological soil crusts to raindrop erosivity and underlying influences in the hilly Loess Plateau region, China. Biodiversity and conservation, 23(7), 1669-1686.
